# Do the Reasons Emerging Adults Become Informal Caregivers Relate to Future Willingness to Care? A Mixed-Methods Study

**DOI:** 10.1093/geroni/igaa057.1149

**Published:** 2020-12-16

**Authors:** Anastasia Canell, Hannah Bashian, Grace Caskie

**Affiliations:** Lehigh University, Bethlehem, Pennsylvania, United States

## Abstract

Approximately 12-18% of family caregivers to older adults in the U.S. are emerging adults (aged 18-25), yet minimal research focuses on this subgroup of caregivers (Levine, 2005). Although several theories have developed to explain the growing number of emerging adults assuming informal caregiving roles (e.g., alleviating burden on middle-aged caregivers, family obligation; Dellmann-Jenkins & Brittain, 2003), the reasons why emerging adults become caregivers have not been studied. In the current study, a sample of 248 emerging adult caregivers were asked to describe the circumstances that led to providing unpaid care to an older adult. Using discovery-oriented coding methodology, 11 themes emerged within participants’ responses: care-recipient illness (35.5%), familial relationship (35.5%), care-recipient became dependent (23.8%), proximity (13.7%), only option (10.1%), reciprocal care (8.9%), availability (8.5%), age-related decline (6.9%), monetary restrictions (6.9%), care-recipient desire (6.0%), and community service (4%). Follow-up analyses found that participants who identified familial relationships (e.g., “They are my parents so I felt obligated...”) were less likely to endorse willingness to provide nursing care in the future compared to those who did not identify familial relationships (p=.032). Participants who identified care-recipient dependency as a circumstance leading to caregiving (e.g., “My grandmother fell and was in rehab...”) were more likely to endorse willingness to provide instrumental (p=.034) and emotional (p=.047) care in the future than those who did not identify care-recipient dependency. These results demonstrate the unique reasons that may lead emerging adults to begin caregiving and how these reasons relate to future willingness to care for an older adult.

